# Development and validation of a clinical score for early diagnosis of constipation in critically ill children

**DOI:** 10.1038/s41598-023-41674-5

**Published:** 2023-09-08

**Authors:** J. López, C. Sánchez, S. N. Fernández, R. González, M. J. Solana, J. Urbano, J. López-Herce

**Affiliations:** 1 Pediatric Intensive Care Department, Hospital General Universitario Gregorio Marañón, Instituto de Investigación Sanitaria Gregorio Marañón, Primary Care Interventions to Prevent Maternal and Child Chronic Diseases of Perinatal and Development Origin Network (RICORS) RD21/0012/0011 of Instituto de Salud Carlos III, Complutense University of Madrid, Spain. C/ Dr Castelo 47, 28009 Madrid, Spain; 2 Pediatric Gastroenterology Unit, Hospital General Universitario Gregorio Marañón, Instituto de Investigación Sanitaria Gregorio Marañón, Primary Care Interventions to Prevent Maternal and Child Chronic Diseases of Perinatal and Development Origin Network (RICORS) RD21/0012/0011 of Instituto de Salud Carlos III, Complutense University of Madrid, Madrid, Spain

**Keywords:** Gastroenterology, Risk factors

## Abstract

Constipation affects almost 50% of critically ill pediatric patients and is related to their morbidity and mortality. However, little attention is paid to it and it is diagnosed late and when there are already complications. The objective of this study is to develop and validate a score to identify critically ill children with high risk of constipation 48 h after admission. A single center two phase-study was carried out; the first one (retrospective observational study) to develop the score and the second one to validate it in another prospective observational study. Children between 15 days of life and 18 years old admitted to the PICU for more than 3 days were included. Demographic and clinical data during the first 48 h after PICU admission were collected. Univariate and multivariate analysis and ROC curves were used to develop and validate the score. Data from 145 patients (62.8% boys) with a mean age of 34.9 ± 7.3 months were used to develop the score. Independent factors identified to develop the score were: weight > 7 kg, admission to PICU after surgery, need of vasoconstrictors, doses of fentanyl ≥ 2 mcg/kg/h, and initiation of enteral nutrition later than 48 h after admission. Two cut-off values were identified to set low constipation risk (< 5.7 points) and high constipation risk (> 6.2 points). This score was validated in 124 patients showing a sensibility of 63.2%, specificity of 95.5% and a positive/negative predictive values (P/NPV) of 100% and 82.1% respectively to identify constipated patients. This is the first score to identify high constipation risk in critically ill children. This score is easy to apply, and internal validation has shown a PPV of 100%.

## Introduction

Within the last two decades, health professionals have become aware of the importance of constipation in Intensive Care Units (ICU), and constipation is now perceived to be increasingly important^1–5^. However, no standard definition has been universally accepted (children or adults)^1,2,5–7^. Rome criteria^8,9^ are not valid for critically ill patients because in these patients, constipation is a multifactorial disorder frequently related to sedative and vasoconstrictor drugs^1,10–12^, hypotension^13^ and immobility^14^, not a functional one^15^. This lack of definition is one of the main obstacles to reach an early diagnosis.

Besides, constipation diagnosis is frequently delayed due to it is considered a less severe complication in critically ill patients^4,16^ compared to hemodynamic, respiratory, or renal problems. However, constipation has been related in critically ill adult patients to delirium^17^, higher illness clinical severity^13,18^, longer duration of mechanical ventilation (MV)^13^, ventilator weaning^19^, ICU and hospital length of stay^3,5,10,13,20,21^ and higher hospital costs^5^. In critically ill children has been related to higher illness clinical severity^1^. Frequently, diagnosis is delayed until constipation is well established and complications due to constipation are already present. At this point, treatment is more difficult because of a lack of tolerance to EN and because some treatments are contraindicated when ileus is present^20,22^.

In pediatric patients, we found that 46.7% of children admitted to the Pediatric Intensive Care Unit (PICU) suffered from constipation^1^. Some independent risk factors for constipation were identified in this population: higher body weight, higher severity of illness scores, admission after surgery and treatment with vasoconstrictors^1,6^. In critically ill adults, sedatives, admission after surgery and late enteral nutrition (EN) were identified as independent risk factors for constipation^3^.

The objective of this study was to elaborate an easy clinical score from our previous data that could be applied at 48 h after admission, to identify children with high risk of constipation and to validate it in a similar population.

## Methods

We used data from a previous single center study designed to describe epidemiological factors of constipation in critically ill children followed up to 30 days or the PICU discharge^1^. With this data, we developed a score which was capable to detect high and low constipation risk just 48 h after PICU admission. After that, we prospectively obtained another sample in the same centre for 1 year to validate our score. Our PICU is a mixed unit (medical and surgical) with 400–450 annual admissions, of which almost 50% are postoperative cardiac surgery.

Inclusion criteria were children between 15 days of life and 18 years old admitted to the PICU for more than 3 days and whose parents or legal guards signed the consent form. Children with gastrointestinal disease presented prior to admission were excluded. Children from the first study with missing data during the first 48 h after admission and those children with constipation treatment before constipation definition was settled in the second study, were also excluded. For those children re-admitted 24 h after PICU discharge, only the first PICU admission was considered.

Constipation was defined as absence of defecation for more than 3 days after PICU admission^1,6,16,19^. Data analyzed included age, sex, weight, diagnosis and illness severity scores at admission: Pediatric Risk of Mortality III (PRISM III), Pediatric Index of Mortality 2 (PIM2), and Pediatric Logistic Organ Dysfunction (PELOD)^23–25^, length of PICU stay, and mortality. Data during the first 48 h after admission of midazolam, fentanyl, muscle relaxant, and inotropic agents (epinephrine or norepinephrine) administered, as well as the need for invasive or non-invasive mechanical ventilation and daily volume of EN were also analyzed. EN is started, increased or decreased and delivered according to a standard protocol followed by every physician. No medications to encourage bowel movements were administered in the first 48–72 h after admission.

Data analysis was performed using the SPSS 21.0 software package (IBM SPSS Statistics, Chicago). Continuous variables are expressed as mean ± standard deviation (SD) and categorical variables as percentages. Kolmogorov–Smirnov test was used to check normality. Comparisons of continuous and categorical variables were performed using the T or χ^2^ test (Fisher exact test when expected frequency was < 5) respectively. Univariate analysis was initially performed to identify factors associated with constipation. ROC curves were used to establish the best cutoff values for continuous variables. Results are expressed as odds ratio (OR) with 95% confidence intervals (95% CI). Multivariate analysis was then performed using a predictive logistic backward regression model that included those variables with statistical significance in the univariate analysis to identify independent factors. With these independent factors, a clinical score was developed: each OR value was divided by the lower OR obtained in the multivariate analysis and rounded to the nearest one decimal number. ROC curves were used to assure that this approach was correct and to establish the more sensitive and specificity cutoff values for the score. Statistical significance was taken as a p value of less than 0.05.

### Ethics approval and consent to participate

Both studies were approved by the local Institutional Review Body of the Hospital General Universitario Gregorio Marañón. These studies were performed in accordance with the ethical standards laid down in the 1964 Declaration of Helsinki and its later amendments. Informed consent was obtained from all subjects (their parents or legal guards) involved in the study.

## Results

### Score development

One hundred and forty-five patients (62.8% boys) with a mean age of 34.9 ± 7.3 months were studied. Surgical admissions accounted for 59.3% with 84 (97.6%) of surgical admissions from cardiac surgery. During the first 48 h after admission, 87 (60%) of patients were on MV and 100 (69%) had already started EN. Vasoconstrictors (epinephrine and/or norepinephrine) were required in 40 patients (27.6%) and 29 (20%) needed neuromuscular blockers. Midazolam and fentanyl were the most used sedatives; in 83 (57.2%) and 79 (54.5%) of patients respectively.

In the univariate analysis, factors more associated (OR > 3 and *p* < 0.06) with constipation were: weight > 7 kg, admission to PICU after surgery, risk of mortality according to PIM 2 > 4.5% at PICU admission, need of vasoconstrictors, doses of fentanyl ≥ 2 mcg/kg/h and initiation of EN more than 48 h after admission (Table 1). In the multivariate analysis, PIM 2 score was a dependent factor while the other five factors proved to be independently associated with constipation, and the score was elaborated with them (Table 1).

**Table 1:** Univariate and multivariate analysis of the variables (during first 48 h after admission) associated with constipation and score of constipation.

Variable	Univariate analysis		Multivariate analysis		Score
	OR	95% CI	OR	95% CI	Score^a^
Demographic data at admission					
Weight > 7 kg	**11.55**	**2.55–52.33**	**13.07**	**4.47–38.19**	13.07/2.73 ≈ **4.8**
Postoperative	3.14	0.97–10.14	**2.73**	**1.06–7.04**	2.73/2.73 = **1**
Severity scores					
PRISM III > 3%	0.42	0.10–1.74			
PELOD > 1.2%	1.65	0.46–5.90			
PIM 2 > 4%	**3.19**	**1.09–9.38**	2.36	0.90–6.18	Not included
Vasoconstrictors (Epinephrine/Norepinephrine)	**9.84**	**1.77–54.50**	**8.37**	**2.44–28.78**	8.37/2.73 ≈ **3**
Mechanical ventilation	0.61	0.13–2.82			
Non-invasive ventilation	0.63	0.20–1.99			
Sedation/analgesia					
Midazolam (≥ 2 mcg kg^−1^ min^−1^)	0.35	0.05–2.31			
Fentanyl (≥ 2 mcg kg^−1^ min^−1^)	**13.85**	**1.68–113.91**	**3.68**	**1.38–9.85**	3.68/2.73 ≈ **1.3**
Muscle relaxants (vecuronium)	0.94	0.18–4.89			
Initiation of EN > 48 h	**3.71**	**1.12–12.23**	**3.45**	**1.22–9.71**	3.45/2.73 ≈ **1.2**

OR: Odds Ratio. 95% CI: 95% confidence interval. PRISM III: Pediatric Risk of Mortality III. PELOD: Pediatric Logistic Organ Dysfunction. PIM 2: Pediatric Index of Mortality. EN: Enteral nutrition.

*p* < 0.05 are marked in bold.

^a^ Number of points assigned for each variable if the patient meets the condition during first 48 h of admission.

**Figure 1:**
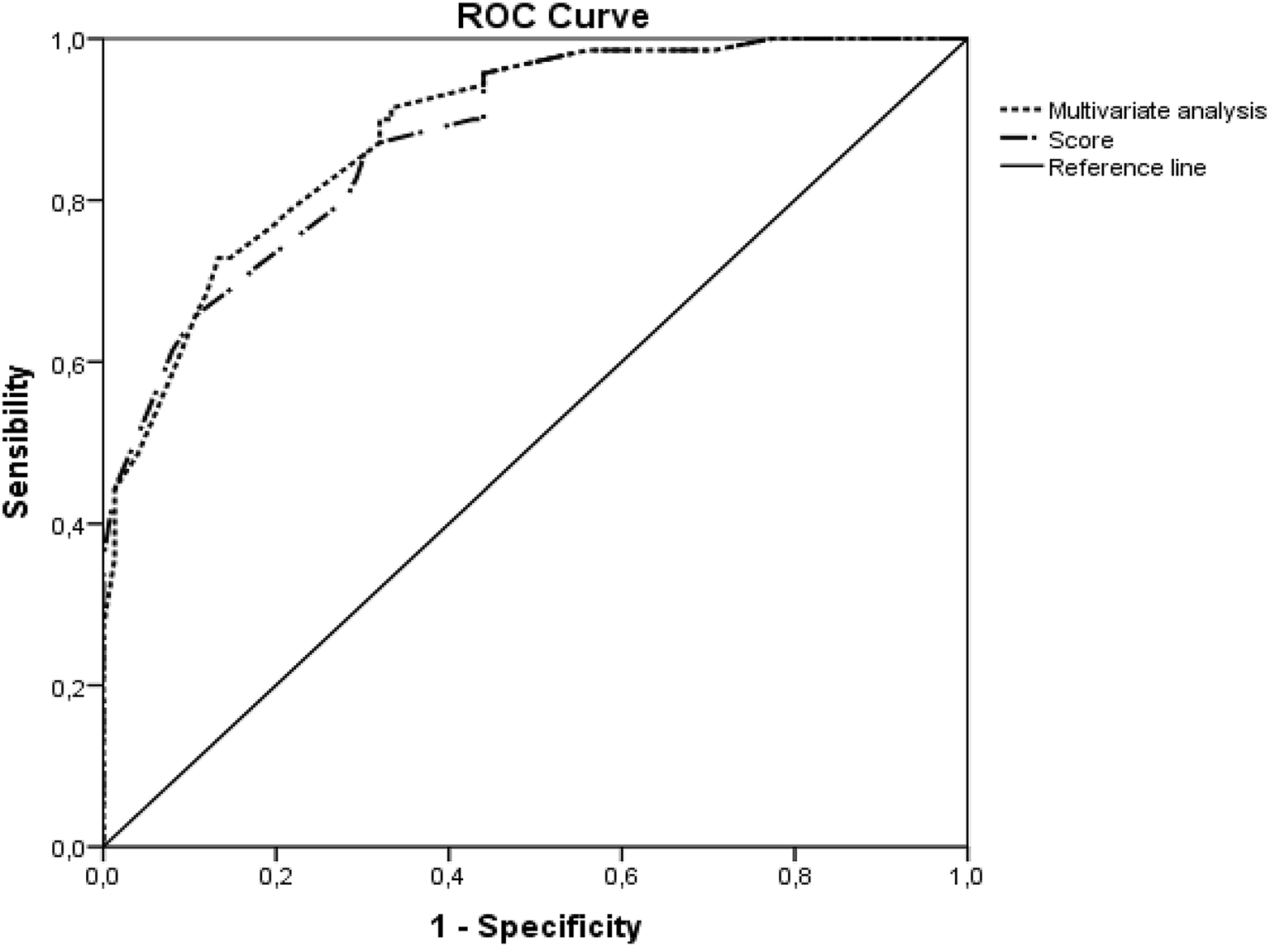
ROC curve comparing multivariate analysis and the constipation score.

This score showed an area under the curve (AUC) of 0.878 with a 95% CI of 0.825–0.931 (*p* < 0.001) very close to the AUC of the complete multivariate analysis, 0.886 with a 95% CI of 0.835–0.938 (*p* < 0.001) (Fig. 1). Two cut-off values with good sensibility and specificity according to the ROC curve were identified to set low constipation risk (< 5.7 points) and high constipation risk (> 6.2 points) (Table 2).

**Table 2:** Sensibility, specificity and positive and negative predictive values of the constipation score cut off values.

Cut off values	< 5.7 points Low constipation risk	> 6.2 points High constipation risk
Statistics		
Sensibility (95% CI)	80% (69.1%-87.8%)	65.7% (54%-75.8%)
Specificity (95% CI)	72% (60.9%-80.9%)	89.3% (80.1%-94.7%)
Positive Predictive Value (95% CI)	72.7% (61.8%-81.5%)	85.2% (73.1%-92.5%)
Negative Predictive Value (95% CI)	79.4% (68.2%-87.4%)	73.6% (63.7%-81.6%)

95% CI: 95% confidence intervals.

### Score validation

One hundred and twenty-three patients (56.1% boys) with a mean age of 37.5 ± 4.9 months were studied and followed up until the fourth day of PICU admission. Surgical admissions accounted for 43.9%. Median length of PICU stay was 12.6 ± 1.5 days and 8 patients (6.5%) died. During the first 48 h after admission, 58 (47.2%) of patients were on MV, 25 (20.3%) required vasoconstrictors and 91 (74%) had already started EN. Midazolam and fentanyl were also the most used sedatives in 34 (27.6%) and 37 (30.1%) of patients respectively and neuromuscular blockers were required in 16 patients (13%). Comparison between both populations is in Table 3.

**Table 3:** Comparison between both populations.

Variables	Score development N = 145	Score validation N = 123	*p*
Demographic data at admission			
Age (months)	34.9 ± 3.7	37.5 ± 4.9	0.676
Weight (kg)	13.5 ± 1.2	13.3 ± 1.2	0.882
Male	91 (62.8%)	69 (56.1%)	0.268
Postoperative	86 (59.3%)	54 (43.9%)	**0.012**
PIM 2 (%)	12 ± 1.6	7.1 ± 1.4	**0.024**
Continuous renal replacement therapy	21 (14.5%)	7 (5.7%)	**0.009**
Extracorporeal membrane oxygenation	16 (11%)	4 (3.3%)	**0.008**
Vasoconstrictors (Epinephrine/Norepinephrine)	40 (27.6%)	25 (20.3%)	0.167
Mechanical ventilation	87 (60%)	58 (47.2%)	**0.035**
Non-invasive ventilation	54 (37.2%)	45 (36.6%)	0.912
Sedation			
Midazolam (≥ 2 mcg kg^−1^ min^-1^)	83 (57.2%)	34 (27.6%)	** < 0.001**
Fentanyl (≥ 2 mcg kg^−1^ min^-1^)	79 (54.5%)	37 (30.1%)	** < 0.001**
Muscle relaxants (vecuronium)	29 (20%)	16 (13%)	0.127
Initiation of EN > 48 h	45 (31%)	32 (26%)	0.366
Length of PICU stay (days)	24,4 ± 5,6	12.6 ± 1.5	**0.045**
Mortality	8 (5.5%)	8 (6.5%)	0.734
Constipation	70 (48.3%)	57 (46.3%)	0.752

PIM 2: Pediatric Index of Mortality 2. EN: enteral nutrition; PICU: pediatric intensive care unit.

*p* < 0.05 are marked in bold.

Fifty-seven (46.3%) patients developed constipation during the first 4 days after PICU admission. According to the scale developed, two days after PICU admission, 37 patients would have been pointed as high constipation risk and these 37 patients finally developed constipation (Table 4).

**Table 4:** Constipation score validation.

Score 48 h after PICU admission	Clinical situation	
	Non constipated	Constipated
	N = 66	N = 57
Low constipation risk (< 5.7 points)	63	15
Negative Predictive Value (95% CI)	**80,8% (70.5%-88.1%)**	19.2%
Specificity (95% CI)	**95,5% (86.9%-98.9%)**	26.3%
Intermediate constipation risk (5.7–6.2 points)	3	5
High constipation risk (> 6.2 points)	0	37
Positive Predictive Value (95% CI)	0%	**100% (88.8%-100%)**
Sensibility (95% CI)	0%	**64,9% (51.9%-76%)**

PICU: pediatric intensive care unit; 95% CI: 95% confidence intervals.

*p* < 0.05 are marked in bold.

## Discussion

Constipation in is associated with important complications and high mortality rate in critically ill patients^1,3,5,6,10,13,16–21^. However, until now, very few pediatric studies about constipation in critically ill children have been published^1,2,6^. This is the first study that have developed a simply score to early diagnose this complication.

A very important barrier to performing epidemiology and treatment studies is the lack of universal diagnostic criteria for constipation^1,2,7,16,18,26^. International consensus about this item is focused in functional gastrointestinal disorders but there is no consensus about secondary constipation^8,9,27^. Moreover, constipation in critically ill patients has multiple origins, including clinical situations, drugs and environmental circumstances and is not limited to only one factor in each critical patient^15,28^.

Several constipation management protocols in critically ill adults have been proposed^4,10,29,30^ and even a risk assessment scale^31^ but no early diagnostic score has been developed.

Some studies in critically ill adult patients have shown the utility of protocols to identify and treat constipation early^32–34^ but there is nothing similar in the pediatric population. We have developed a simple score which can identify critically ill children with low and high risk of constipation development 48 h after PICU admission. This information could be used to start the treatment in high-risk patients early and to avoid delay in diagnosis and treatment.

Our score is based in five factors: weigh and vasoconstrictors are the most important. Older children are continent, so immobilization, a non comfortable environment and inappropriate bathrooms make it very difficult for them to maintain regular bowel movements during PICU admission^1,14,35^. However, this factor probably is not so important in severely ill patients with higher doses of sedatives.

The importance of vasoconstrictors could be more related to the hemodynamic and general situation (hypotension, gastrointestinal hypoperfusion and severity of illness) than to the direct effect of these drugs on the bowel motility^1,10,11,13,21^. Higher dosages of fentanyl are directly related to their peripheral action over mu opioid receptors, which decreases intestinal motility^1–3,10,12^. Surgery could influence constipation because of the effect of anesthetic drugs on the bowel motility. Finally late EN have been also identified as constipation risk factors in critically ill adults^3,16^. EN is one of the most important factors that induces bowel motility and prevents constipation.

ROC curves and AUC of multivariate analysis and score adjustment in our study were almost equal, so mathematical rounding did not change their predictive capability. The lower limit of our score showed a sensibility of 80% and it can identify non constipated patients with 79.4% reliability (negative predictive value), while the upper limit showed a specificity of 89.3% with a capacity to identify constipated patients of 85.2%.

Regarding the population collected for the scale validation, it was similar but not identical to the reference one. This new group of patients seemed to be slightly less severe since their PIM 2 scale score, length of PICU stay, need for MV, and therefore treatment with midazolam and fentanyl, continuous renal replacement therapy and extracorporeal membrane oxygenation was significantly lower than reference population values. However, the independent factors used to create the constipation risk scale were similar between both populations, especially those with more specific weight (weight and need for vasoconstrictors), except for the need of fentanyl. For this reason, we consider that the selected population was adequate for the internal validation of the constipation risk scale.

The fact that the percentage of constipation is practically identical between both populations, although they are different in some risk factors such as opiates, clinical severity, PICU stay or MV, supports the hypothesis that constipation in the critical child, is an entity with a multifactorial cause in which various factors act synergistically^15,16,19,36–39^.

However, our population may not be representative of children admitted to other PICUs, since a high percentage of patients with heart disease and postsurgical patients enter our population. For this reason, studies are necessary to confirm the utility of this scale in other PICUs.

The developed scale has shown, on a different population, an excellent ability to identify patients at high risk of constipation 48 h after PICU admission. This scale is simple, easy and quick to apply. With this information, the initiation of early treatment in these high-risk patients could be considered, trying to reduce complications due to delayed diagnosis and treatment. Two studies in critical adults have raised this possibility. Guardiola et al. showed how early treatment on the second day of ICU admission in mechanically ventilated patients was better than conventional treatment administered after diagnosing constipation^32^. Masri et al. also reached a similar conclusion but with a treatment just upon ICU admission^33^.

In contrast, the moderate sensitivity (64.9%) presented by our scale, means that a few patients who will eventually develop constipation, are not identified 48 h after admission. Regarding the patients who will not develop constipation, the score has a high specificity and negative predictive value also. Therefore, in low-risk patients (< 5.7 points), no intervention is necessary, but a follow up is necessary, since up to a quarter of them may be constipated at the end.

Considering that, at present, there is no other method to make an early diagnosis of constipation in critically ill children, we consider this scale is useful to identify patients with high risk of constipation. Moreover, the use of this scale can increase awareness and active surveillance of this problem by improving clinical practice and can serve as a basis for comparing risk groups and assessing the effect of various treatments that could be applied early.

Several limitations in this study should be considered. First, this is a single-centre study in a mixed PICU with a relatively low number of patients. Second, the development of the constipation risk scale, is based on pre-existing data used for another study, although the objective of both studies was similar. However, the fact that the population used for its validation was collected prospectively, this limitation can be solved. And finally, the fact that in our unit we use midazolam and fentanyl as the first line of treatment and during the first 5 days of PICU admission, means that we have developed this score according to these drugs without taking into account other sedoanalgesics or the opioid/morphine equivalent, which may limit its application in other units.

## Conclusions

This is the first tool to improve constipation diagnosis in critically ill children. Our score is easy to use and can identify high risk of constipation development in our critically ill children. Internal validation has been performed but external validation should be carried out to assure its utility through further future multicenter studies. This score also opens a new line of investigation related to prophylactic treatments for constipation in critically ill children.

## Data Availability

The datasets used and/or analysed during the current study are available from the corresponding author on reasonable request.

## Abbreviations

AUC: Area under the curve
EN: Enteral nutrition
ICU: Intensive care unit
MV: Mechanical ventilation
OR: Odds ratio
PELOD: Pediatric logistic organ dysfunction
PICU: Pediatric intensive care unit
PIM 2: Pediatric index of mortality
PRISM III: Pediatric risk of mortality III
SD: Standard deviation
95% CI: 95% confidence interval
